# Downregulation of miR-17~92 Expression Increase Paclitaxel Sensitivity in Human Ovarian Carcinoma SKOV3-TR30 Cells via BIM Instead of PTEN

**DOI:** 10.3390/ijms14023802

**Published:** 2013-02-08

**Authors:** Ting Shuang, Chunxue Shi, Shuang Chang, Min Wang, Cui Hong Bai

**Affiliations:** 1 Department of Obstetrics and Gynecology, Shengjing Hospital of China Medical University, Shenyang 110004, China; E-Mails: shuangting87@163.com (T.S.); c.shuang-0519@163.com (S.C.); myearth126@126.com (C.H.B.); 2 Shenyang Maternity and infant Hospital, 90 Qingnian Road, Shenhe District, Shenyang 110014, China; E-Mail: shichunxue_502@163.com

**Keywords:** miR-17~92 cluster, transduction, ovarian carcinoma, paclitaxel resistance, PTEN, BIM

## Abstract

To better understand the molecular mechanisms of paclitaxel resistance in ovarian carcinoma, we evaluated the expression of miRNAs using miRNA microarray between human ovarian carcinoma SKOV3 cells and paclitaxel resistant SKOV3-TR30 cells. Results showed that 69 miRNAs were upregulated while 102 miRNAs were downregulated in SKOV3-TR30 cells. Using real-time PCR, we further clarified that miR-17~92 was overexpressed in SKOV3-TR30 cells compared with that in SKOV3 cells. We then established stable virally transduced SKOV3-TR30-m-PTIP-Sponge all SKOV3-TR30 cells and its vector-only control SKOV3-TR30-m-PTIP-GFP cells. Real time-PCR revealed that SKOV3-TR30-m-PTIP-Sponge all cells expressed approximately 6.18-fold lower levels of miR-17~92 compared with the control group. Decreased expression of miR-17~92 resulted in cell cycle arrest in the G2/M phase and growth inhibition. After the transduction, the BIM protein level was increased in SKOV3-TR30 cells and luciferase reporter assays revealed that miR-17~92 binds directly to the 3′-UTR of BIM. Results of luciferase reporter assays accompanied with Western Blot showed that although miR-17~92 binds directly to the 3′-UTR of PTEN, the PTEN protein expression level was upregulated slightly while the result is of no statistical significance. Our results showed that miR-17~92 could be a causal factor of the downregulation of BIM in SKOV3-TR30 cells and thus induce the paclitaxel resistance in SKOV3-TR30 cells.

## 1. Introduction

Ovarian carcinoma is one of the most common gynecological malignant tumors. Its incidence is ranked second in malignant tumors of the female reproductive system and is gradually increasing in recent years. The most recommended treatment worldwide is surgery, along with paclitaxel and platinum–based (first-line chemotherapy) adjuvant chemotherapy; however, its mortality rate is still about 70%, which is the highest in gynecological malignancies [1]. One of the most significant reasons for such high morality rate is that about 30%~40% of patients with ovarian carcinoma are resistant to chemotherapy and, moreover, 60% of first-line chemotherapy-sensitive patients are resistant to chemotherapy after six months. Therefore, clarification of the exact mechanism of resistance and resistance reversal of ovarian carcinoma has become extremely urgent and important research topic.

MicroRNAs (miRNAs) contain 18–24 nucleotides and are small, non-coding RNAs that post transcriptionally regulate gene expression through translational repression and mRNA degradation [2]. Recent research has shown the tumor suppressant and oncogenic potential of a number of miRNAs, underscoring their importance in human cancer therapeutic and diagnostic applications [3–7].

BIM is a member of the BH3-only family of pro-apoptotic proteins and is expressed in a wide variety of tissues. BIM can initiate apoptosis by directly activating Bax through interaction with the Bcl-2/Bax herterodimer complex, which can further induces mitochondrial cell death [8,9]. It plays a critical role in tumor cell biology, including the regulation of tumorigenesis through activities as a tumor suppressor, tumor metastasis, and tumor cell survival. Therefore, it has gradually become an interesting target for cancer chemotherapy. PTEN plays a well-established role in the negative regulation of the PI3K pathway, which is frequently activated in several cancer types, including ovarian cancer. PTEN loss of function occurs in a wide spectrum of human cancers through mutations, deletions and transcriptional silencing. The expression of BIM and PTEN is highly regulated by its transcriptional and post-translational levels.

Overexpression of miR-17~92 has been observed in lymphomas and solid tumors [10] and is related to cell proliferation. The gene cluster of miR-17~92 resides with intron 3 of cl3orf25 non-protein-coding gene at 13q31.3 gene [8,11]. Studies by Lewis, BP, and others [12] have shown that miR-17~92 may play a role in PTEN and BIM. In recent years, more and more studies have reported that abnormal expression of PTEN and BIM has participated in the formation of tumor drug-resistance [13–15].

Yet it is not clear if miR-17~92 gene cluster has an impact on the paclitaxel resistance of ovarian carcinoma through affecting the expression of the BIM or PTEN protein. Furthermore, the effect of both BIM and PTEN on paclitaxel resistant in ovarian cancer cells has not been thoroughly researched; particularly, the mechamism involved in their expression regulation has barely been studied in ovarian carcinoma chemoresistance.

In this study, we investigated whether BIM or PTEN gene was post-transcriptionally regulated by miR-17~92 and the contribution of miR-17~92 to BIM or PTEN protein levels in SKOV3-TR30 cells. In addition, we also investigated the impact of miR-17~92 on SKOV3-TR30 cell proliferation and cell cycle. We aim to get a better understanding of the molecular mechanisms of paclitaxel resistance in SKOV3-TR30 cells, to provide a clue for further investigation of the function of miR-17~92 and its target genes, and their correlation in ovarian carcinoma paclitaxel resistance.

## 2. Results

### 2.1. MiRNA Expression Profiling

**Figure 1:**
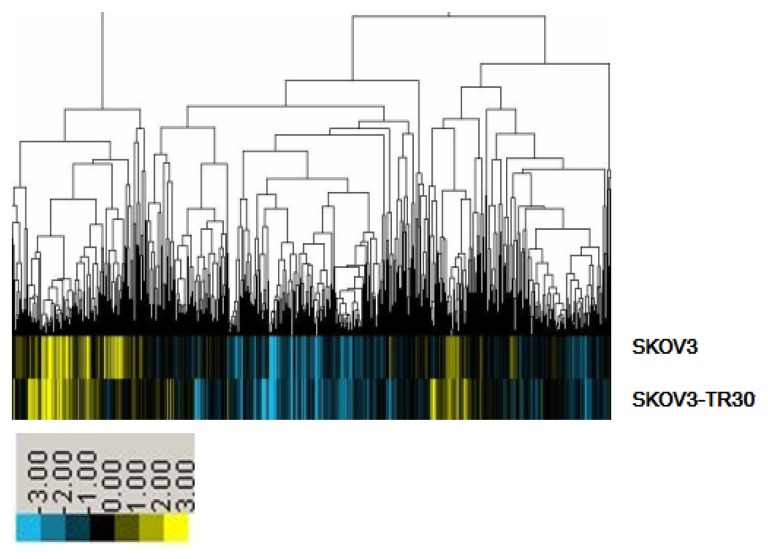
Analysis of miRNAs Expression Profile of Sensitive and Drug-resistant Human Ovarian carcinoma Cells. Data were analyzed by the CLUSTER analysis software CLUSTER 3.0. Yellow means high expression, blue represents the low expression.

A microarray platform was used to analyze and compare the pattern of miRNA expression between the parental SKOV3 cell line and its counterpart paclitaxel resistant SKOV3-TR30 cell line. The expression profiles of 171 miRNAs changed significantly: 69 upregulated miRNAs (miR-17, miR-19b, miR-92-1) and 102 downregulated miRNAs (miR-134, miR-34, miR-196b) in SKOV3-TR30 cells as compared with SKOV3 cells (Figure 1).

### 2.2. miR-17~92 Was Overexpressed in SKOV3-TR30 Cells Compared with SKOV3 Cells

**Figure 2:**
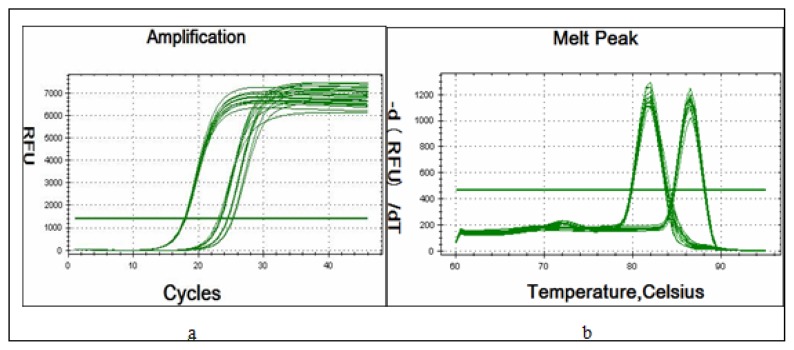
Amplification Curve and Melting Curve of miR-17~92 Expression in SKOV3, SKOV3-TR30, SKOV3-TR30-m-PTIP-Sponge all cells and the SKOV3-TR30-m-PTIP-GFP cells Detected by real-time PCR. (**a**) Amplification Curve of Expression of miR-17~92; (**b**) Melting Curve of Expression of miR-17~92.

We next studied the expression level of miR-17~92 in SKOV3 and SKOV3-TR30 cells using real-time PCR. Amplification Curve and Melting Curve of miR-17~92 and internal control β-actin see Figure 2. The expression level of miR-17~92 in the SKOV3-TR30 cells is indicated as “1” as control group. Compared to SKOV3-TR30 cells, the expression level of miRNA-17~92 in SKOV3 cells is 0.557, and the difference is of statistically significant (*t* = 9.193, *p <* 0.05) (Table 1).

The result revealed that the expression level of miR-17~92 was markedly increased in paclitaxel resistant SKOV3-TR30 cells compared with paclitaxel sensitive SKOV3 cells.

### 2.3. Comparison of miR-17~92 Expression Levels in SKOV3-TR30-m-PTIP-Sponge all and the SKOV3-TR30-m-PTIP-GFP

**Figure 3:**
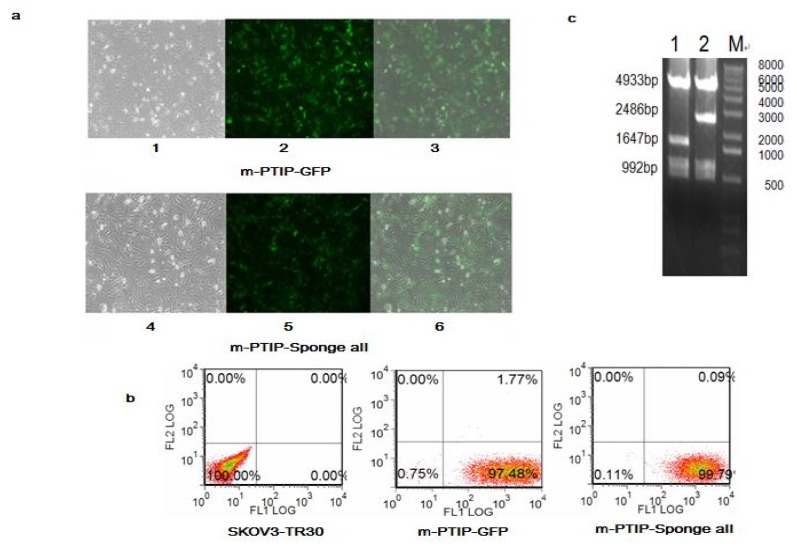
(**a**) Determination of stable virally transduced SKOV3-TR30-m-PTIP-Sponge all cell line and its vector-only control SKOV3-TR30-m-PTIP-GFP by Fluorescence Microscope. (100×) 1, 2, 3: transduced by m-PTIP-GFP empty plasmid 1, white field, 2, with fluorescence, 3, the integration of 1 and 2. 4,5,6: transduced by m-PTIP-Sponge all inhibit plasmid, 4, white field, 5, with fluorescence, 6, the integration of 4 and 5; (**b**) Determination of stable virally transduced SKOV3-TR30-m-PTIP-Sponge all cell line and its vector-only control SKOV3-TR30-m-PTIP-GFP using Flow Cytometry. Ratio of cells expression green fluorescent protein for SKOV3-TR30, SKOV3-TR30-m-PTIP-GFP and SKOV3-TR30-m-PTIP-Sponge all were 0.00, 97.48% and 99.79%; (**c**) Plasmid Restriction Enzyme Digestion Results 1: m-PTIP-GFP empty plasmid, 2: m-PTIP-Sponge all inhibit plasmid, M: Marker.

In order to determine whether the resistance to paclitaxel could be caused by miR-17~92 in SKOV3-TR30, we established stable virally transduced SKOV3-TR30 cell line SKOV3-TR30-m-PTIP-Sponge all cells and its vector-only control SKOV3-TR30-m-PTIP-GFP cells. The m-PTIP-Sponge all inhibit plasmids were identified using restriction enzyme digestion shown in (Figure 3c). Using Flow Cytometry, the purity of stable transfectants identified by the expression of plasmid green fluorescent protein was greater than 95% (Figure 3a,b). We next studied the expression level of miR-17~92 in SKOV3-TR30 -m-PTIP-GFP cells and SKOV3-TR30-m-PTIP-Sponge all cells using real-time PCR. Real-time PCR results revealed that SKOV3-TR30-m-PTIP-Sponge all expressed approximately 6.18-fold lower levels of miR-17~92 (Table 1) compared with which in SKOV3-TR30-m-PTIP-GFP cells.

Amplification Curve and Melting Curve of miR-17~92 and internal control β-actin see Figure 2. The expression level of miR-17~92 in the SKOV3-TR30 cells is indicated as “1” as control. Compared with SKOV3-TR30 cells, the expression level of miRNA-17~92 in SKOV3-TR30-m-PTIP-GFP cells and SKOV3-TR30-m-PTIP-Sponge all cells is 0.507 (*t* = 9.417, *p* < 0.05) and 0.082 (*t* = 23.659, *p* < 0.05), respectively. Contrast the expression level of miR-17~92 in SKOV3-TR30-m-PTIP-GFP cells, which is 0.507 and in SKOV3-TR30-m-PTIP-Sponge all cells which 0.082, the difference is of statistical significance (*t* = 10.726, *p* < 0.05).

### 2.4. Inhibition of miR-17~92 could Inhibit the Proliferation of SKOV3-TR30 Cells

**Figure 4:**
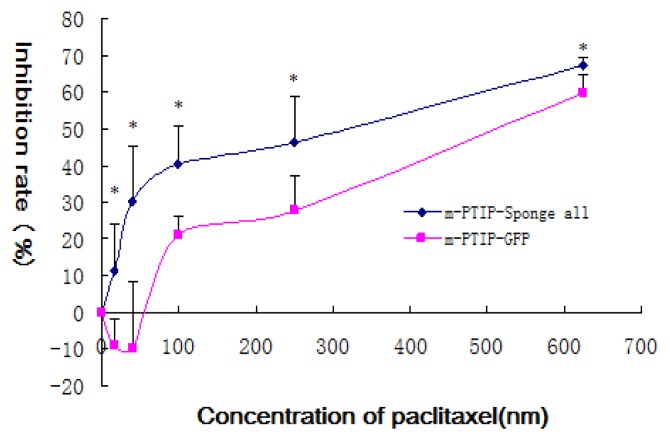
MTT assay was used to detect the effects of miR-17~92 on cell growth in SKOV3-TR30 cells after transduced with either m-PTIP-Sponge all inhibit plasmid or m-PTIP-GFP empty plasmid. Inhibition rate of stable virally transduced SKOV3-TR30-m- PTIP-Sponge all cells and its vector-only control SKOV3-TR30-m-PTIP-GFP cells is of great difference, which has statistical significance (*p* < 0.05). The difference is most obvious when the concentration of paclitaxel is 100 nM (* represents that under the same concentration of paclitaxel, SKOV3-TR30 cells transduced by m-PTIP-Sponge all inhibit plasmid *vs.* SKOV3-TR30 cells transduced by m-PTIP-GFP empty plasmid, *p* < 0.05).

We further study the effects of miR-17~92 expression on the proliferation of SKOV3-TR30 using MTT assay. Paclitaxel inhibited cell proliferation in both SKOV3-TR30-m-PTIP-Sponge all and SKOV3-TR30-m-PTIP-GFP, and with increased concentration of paclitaxel the inhibitory effects was more obvious. Further more, SKOV3-TR30-m-PTIP-Sponge all cells had significantly reduced cell proliferation (*p* < 0.05) compared with SKOV3-TR30-m-PTIP-GFP (Figure 4) and when the concentration of paclitaxel was 100 nm, the inhibition rate difference was most obvious, which is 40.407% of SKOV3-TR30-m-PTIP-Sponge all cells and 20.934% of SKOV3-TR30-m-PTIP-GFP. IC50 of paclitaxel of SKOV3-TR30-m-PTIP-Sponge all cells and SKOV3-TR30-m-PTIP-GFP cells is 470.80 nM and 170.13 nM respectively.

### 2.5. Decreased Expression of miR-17~92 Resulted in Cell Cycle Arrest in the G2/M Phase

**Figure 5:**
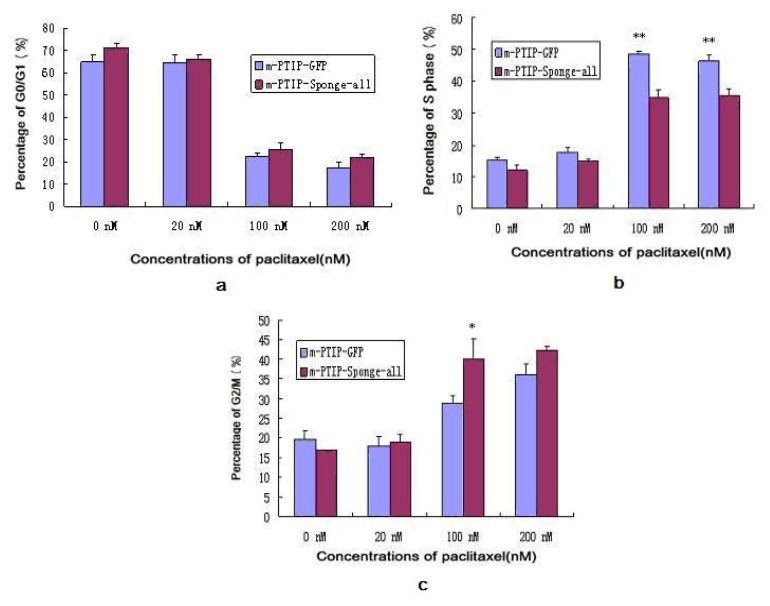
Results of Cell Cycle of SKOV3-TR30 after transduction. (**a**) The Proportion of G0/G1 Phase; (**b**) The Proportion of S Phase; (**c**) The Proportion of G2/M Phase (* respects the group transduced by m-PTIP-Sponge all inhibit plasmid *vs.* the group transduced by m-PTIP-GFP empty plasmid in the same concentration of paclitaxel, *p* < 0.05; ** respects the group transduced by m-PTIP-Sponge all inhibit plasmid *vs.* the group transduced by m-PTIP-GFP empty plasmid in the same concentration of paclitaxel, *p* < 0.01).

We accessed cell cycle distribution profiles after the transduction of SKOV3-TR30 with either miR-17~92-PTIP-Sponge all plasmids or negative control miR-17~92-PTIP-GFP (m-PTIP-GFP) plasmids. Both SKOV3-TR30-m-PTIP-Sponge all and SKOV3-TR30-m-PTIP-GFP cells were treated with paclitaxel (the concentration was 0 nM, 20 nM, 100 nM, 200 nM). Decreased expression of miR-17~92 in SKOV3-TR30-m-PTIP-Sponge all cells resulted in a significant increase in the fraction of cells arrested at G2/M as well as a concomitant decrease in the fraction of cells arrested at S phase compared with SKOV3-TR30-m-PTIP-GFP. These effects were obvious when the concentration of paclitaxel was 100 nm (Figure 5).

### 2.6. BIM, a BH3-only Propoapoptotic Protein, Is a Direct Target of the miR-17~92

**Figure 6:**
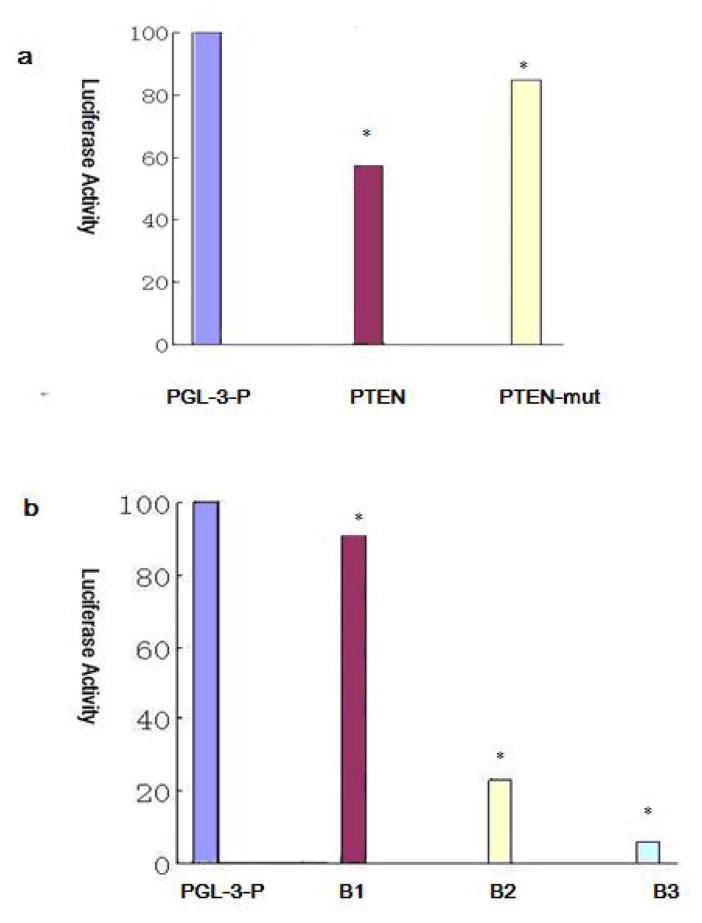
(**a**) Overexpression of miR-17~92 in HEK293 cells inhibited the luciferase activity of the PTEN 3′-UTR reporter plasmid. The 3′-UTR reporter plasmids PTEN or PTEN mut were co-transfected with TMP2-miR-17~92 into HEK293 cells. The activity of firefly luciferase was normalized to that of Renilla luciferase. (**b**) Overexpression of miR-17~92 in HEK293 cells inhibited the luciferase activity of the BIM 3′-UTR reporter plasmid. The 3′-UTR reporter plasmids B1, B2 orB3 were co-transfected with TMP2-miR-17~92 into the HEK293 cells. The activity of firefly luciferase was normalized to that of Renilla luciferase. * *p* < 0.05 compared with the control.

**Figure 7:**
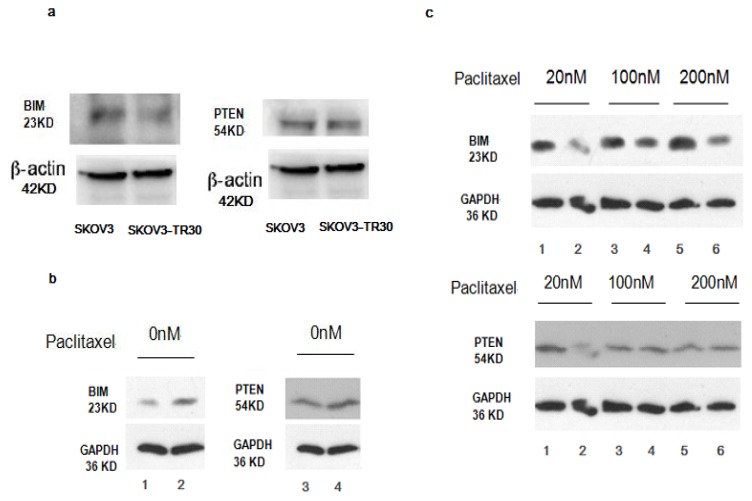
(**a**) The samples from SKOV3 cells and SKOV3-TR30 cells were subjected to Western Blot analysis with a BIM and PTEN antibody while β-actin levels were used as loading control; (**b**, **c**) The samples from SKOV3-TR30-m-PTIP-Sponge all cells and its vector-only control SKOV3-TR30-m-PTIP-GFP cells were subjected to Western Blot with BIM and PTEN antibody while GAPDH levels were used as loading control; (**b**) Decreased expression of miR-17~92 upregulates the expression of BIM instead of PTEN in SKOV3-TR30 cells without treatment of paclitaxel. 1,3 Protein samples from SKOV3-TR30 cells transduced by m-PTIP-GFP empty plasmid. 2,4 Protein samples from SKOV3-TR30 cells transduced by m-PTIP-Sponge all inhibit plasmid; (**c**) Decreased expression of miR-17~92 upregulates the expression of BIM instead of PTEN in SKOV3-TR30 cells after the treatment of paclitaxel with different concentration.1, 3, 5, Protein samples from SKOV3-TR30 cells transduced by m-PTIP-Sponge all inhibit plasmid 2,4,6: Protein samples from SKOV3-TR30 cells transduced by m-PTIP-GFP empty plasmid. The concentration of paclitaxel: 1,2: 20 nM, 3,4: 100 nM, 5,6: 200 nM.

As result shows miR-17~92 expression is significantly higher in SKOV3-TR30 than in SKOV3. To demonstrate the BIM protein level was directly mediated by miR-17~92 through biding to 3′-UTR of BIM, we co-transfected the BIM 3′-UTR (B1,B2,B3) along with TMP2-miR-17~92 into HEK293 cells and than performed luciferase reporter assays. The luciferase reporter plasmid contains a segment of the BIM 3′-UTR, among the three cloned fragments of the BIM 3′-UTR. B2 containing binding sites for miR-17-5p/-20a and miR-92, and B3 contains binding sites for miR-19 and miR-92 while there is not binding site of B1 with miR-17~92. The luciferase activity of the reporter plasmid containing the B1, B2 or B3 was decreased to 91.5%, 23.75% and 6.25% separately compared with the control construct (Figure 6b). We then used SKOV3 and SKOV3-TR30 cells to clarify if miR-17~92 has an effect on BIM protein expression which then trigger paclitaxel resistance in ovarian carcinoma cells. In contrast to SKOV3 cells, SKOV3-TR30 cells shows significantly decreased expression of BIM (Figure 7a, Table 2). We further applied SKOV3-TR30-m-PTIP-Sponge all cells and the control SKOV3-TR30-m-PTIP-GFP cells for a better understanding about the effect of miR-17~92 on BIM protein. Both of the cell lines were treated with paclitaxel (the concentration was 0 nM, 20 nM, 100 nM, 200 nM). In contrast to SKOV3-TR30-m-PTIP-GFP cells, SKOV3-TR30-m-PTIP-Sponge all cells with a significant lower expression of miR-17~92 showed a increase of the BIM protein level both at the steady-state condition without any paclitaxel (Figure 7b, Table 3) and after the treatment with paclitaxel in different concentrations (Figure 7c, Table 3). These findings suggest that miR-17~92 plays an important role in BIM protein expression decrease through the post-transcriptional regulation.

### 2.7. Effect of miR-17~92 on PTEN Expression

In order to clarify whether miR-17~92 has effect on PTEN expression, we constructed a luciferase reporter plasmid containing point mutations in the predicted miRNA binding sites within the PTEN 3′-UTR (PTEN mut). We then performed luciferase reporter assays in HEK293 cells. The luciferase activity of the luciferase reporter gene containing the wild-type PTEN 3′-UTR decreased 42.6% compared with the control construct pGL-3-P, but the decrease was not seen with the luciferase reporter gene PTEN mut (Figure 6a). That means miR-17~92 binds directly to 3′-UTR of PTEN. We then used SKOV3 and SKOV3-TR30 cells to see if there is a different expression of PTEN. In contrast to SKOV3 cells, expression of PTEN protein is decreased in SKOV3-TR30 cells but the result is of no statistical significance (Figure 7a, Table 4). We then use SKOV3-TR30 to make further clarifications. Results showed PTEN protein level is slightly upregulated in SKOV3-TR30-m-PTIP-Sponge all cells compared with SKOV3-TR30-m-PTIP-GFP cells while the difference is of no statistical significance.

To sum up, expression level of PTEN was not effected; even miR-17~92 binds directly to the 3′-UTR of PTEN. In addition, although miR-17~92 expression level was much lower in SKOV3-TR30-m- PTIP-Sponge all cells, there shows no significant increased expression of PTEN protein. These observations suggested that not only miR-17~92 but also other mechanisms may be responsible for the regulation of PTEN expression, including other miRNAs involved in the post-transcription of PTEN.

## 3. Discussion

The current work shows that miR-17~92 cluster expression correlates with paclitaxel resistance of SKOV3-TR30 cells. It’s also shown that BIM instead of PTEN is suppressed by miR-17~92 cluster via direct binding to the BIM 3′-UTR.

In this study, we established a stable virally transduced SKOV3-TR30 cell line SKOV3-TR30-m- PTIP-Sponge all, which expressed approximately 6.18-fold lower levels of miR-17~92 compared with its vector-only control SKOV3-TR30-m-PTIP-GFP, in order to seek an understanding of the molecular mechanisms of paclitaxel resistance with respect to miR-17~92. At first, we approached this issue by obtaining the miRNA differential expression profile between the parental SKOV3 cells and its paclitaxel resistance SKOV3-TR30 cells by using the array-based miRNA assay. Our results showed that 69 miRNAs are upregulated while 102 miRNAs were downregulated in SKOV3-TR30 cells. Among them miR-17~92 expression was significantly upregulated in paclitaxel resistance SKOV3-TR30 cells compared with that in the parental SKOV3 cells. Using real-time PCR we further clarified that miR-17~92 was overexpressed in SKOV3-TR30 cells compared with SKOV3 cells.

A miR-17~92 cluster comprising miR-17, miR-18a, miR-20a, miR-19a, miR-19b, and miR-92-1 is overexpressed in a large fraction of lymphomas [11]. Besides, miR-17~92 was detected as overexpressed also in multiple myeloma [16], breast cancer [17] and osteosarcoma [18]. Ohyashiki [19] states that miR-92a is not only used for the diagnosis of non-Hodgkin’s lymphoma, but also used as an indicator for monitoring the recurrence of non-Hodgkin’s lymphoma after chemotherapy. Other studies show that the tumorigenic effect of miR-17~92 may be due to synergism between its family members [20].

Significant decreased expression of miR-17~92 in SKOV3-TR30 cells by transduction with miR-17~92 inhibitory plasmids (miR-17-92-PTIP-Sponge all) markedly inhibited cell growth and the inhibition rate is most obvious (40.4%, *p* < 0.05) when the concentration of paclitaxel was 100 nm. Decreased expression of miR-17~92 also resulted in cell cycle arrest in the G2/M phase and is most obvious when the concentration of paclitaxel was 100 nm (*p* < 0.05). At that time, the expression of BIM protein was significantly increased. Thus, it is likely that the downregulation of miR-17~92 is closely associated with the sensitivity to paclitaxel through the upregulation BIM. To further clarify whether the BIM protein level was mediated through miR-17~92, we co-transfected the BIM 3′-UTR along with TMP2-miR-17~92 plasmid into HEK293 cells, than performed luciferase reporter assays. The reporter assays revealed that the firefly luciferase activity from HEK293 cells transfected with the reporter gene containing the wild-type B1, B2 or B3 was decreased to 91.5%, 23.75% and 6.25% separately compared with the control construct (Figure 6b). These findings suggest that BIM is likely to be direct target of miR-17~92 and that BIM protein was regulated at the post-transcriptional level in SKOV-TR30 cells.

So far, the research of the influence of miRNA-17~92 on ovarian carcinoma drug-resistance is rarely reported. Using bioinformatics analysis software, we predict that PTEN and BIM are most likely potential target genes of miR17~92. The newest transgenic animal experiments in 2008 shows that miR-17~92 gene clusters down regulate the expression of PTEN and BIM, which are tumor suppressor factors [21]. In recent years, more and more studies have shown that abnormal expression of PTEN and BIM is mostly related to the formation of tumor’s drug resistance [13–15]. There are also studies show down regulation of PTEN and BIM in certain ovarian carcinoma cells with feature of chemoresistance [15,22], besides studies of Lewis and BP [10] show that miR-17~92 takes effect through PTEN and BIM. So in order to further prove whether miR-17~92 cause paclitaxel resistance through PTEN in SKOV3-TR30, we tested the expression of PTEN protein in paclitaxel resistant ovarian carcinoma SKOV3-TR30 cells after transduced with miR-17~92-PTIP-Sponge all. Different from the significant upregulation of BIM protein, PTEN was upregulated slightly while the result is of no statistical significance. The luciferase activity of the reporter gene containing the wild-type PTEN 3′-UTR was decreased 42.6% compared with the control construct pGL-3-P, but the decrease was not seen with the reporter gene PTEN mut (Figure 6a). These observations altogether suggested that PTEN expression is responsible for not only miR-17~92 but also other mechanisms regulating PTEN expression including other miRNAs or other mechanism involved in post-transcription.

## 4. Experimental Section

### 4.1. Cell Culture and Plasmids

The ovarian carcinoma cell line SKOV3 was provided by Tumor Cell Bank Research Institute of the Chinese Academy of Medical Sciences. The paclitaxel-resistant ovarian carcinoma cell line, SKOV3-TR30 with the resistant ability of 27-fold greater than its parental cell line, was derived from SKOV3 cell line and provided by Zhejiang University affiliated Obstetrics and Gynecology Hospital. SKOV3 cells were maintained in McCoy’s 5a medium containing fetal bovine serum with 10%, penicillin with 100 μg/mL and streptomycin with 100 μg/mL. SKOV3-TR30 cells were maintained in McCoy’s 5a medium supplemented with 10% fetal bovine serum, penicillin with 100 μg/mL and streptomycin with 100 μg/mL and paclitaxel with 30 nmol/L, paclitaxel was withdrawn a week before the experiment. All cells were maintained in a humidified atmosphere containing 5% CO_2_ at 37 °C. Cells in the logarithmic phase of growth were used for all studies described. HEK293 cells were maintained in a DMEM medium containing fetal bovine serum with 10% in a humidified atmosphere containing 5% CO_2_ at 37 °C.

MiR-17~92 inhibitory plasmids miR-17~92-PTIP-Sponge all (m-PTIP-Sponge all), empty plasmids miR-17~92-PTIP-GFP (m-PTIP-GFP) and TMP2-miR-17~92 were provided by the Zoology Institute of Chinese Academy of Sciences.

Three plasmids named as B1, B2, B3 are cloned from the 3′-UTR of BIM. These vectors based on the pGL3-P vector were kind gifts from Professor Fu kai. B2 fragment contains miR-17-5p/-20a and miR-92 binding site and B3 fragment contains miR-19 and miR-92 binding site. There is not binding site of B1 with miR-17~92.

PTEN 3′-UTR and PTEN mut 3′-UTR were also gifts from Professor Fu kai. PTEN 3′-UTR contains putative binding sites for both miR20a/17-5p and miR-19.

### 4.2. MicroRNA Gene Chip

We analyze the expression profiles of miRNA on the sensitive and resistant ovarian carcinoma cell lines with using Affymetrix, the Gene Chip^®^ analysis of miRNA chip.

### 4.3. Quantitative Real-Time PCR of miR-17~92 within SKOV3 and SKOV3-TR30 Cells

The expression level of mature miRNAs was determined using the TaqMan real-time quantitative PCR. Briefly, single-stranded cDNA was synthesized from 10ng of total RNA using the TaqMan MicroRNA Reverse Transcription Kit. Each cDNA generated was amplified by quantitative PCR using sequence-specific primers from the TaqMan MicroRNA Assays. PCR primers are as follows: (1) miR-17~92 gene: upstream: 5′-CAGTAAAGGTAAGGAGAGCTCAATCTG-3′, downstream: 5′-CATACAACCACTAAGCTAAAGAATAATCTGA-3′; (2) internal control β-actin gene: upstream: 5′-GCAAAGACCTGTACGCCAACA-3′, downstream: 5′-TGCATCCTGTCGGCAATG-3′. The relative quantity of the target miRNAs was estimated by the 2^−ΔΔCT^ after normalizing to the expression level of β-actin, which was detected by a TaqMan gene expression Assay.

### 4.4. Establishment of Stable SKOV3-TR30 Cell Lines with Induced Expression of miR-17~92 Cluster

To establish miR-17~92-PTIP-Sponge all cell line, the HEK293T cell line was co-transfected with the miR-17~92-PTIP-Sponge all plasmids and pCL packaging plasmid or empty plasmids miR-17~92-PTIP-GFP (m-PTIP-GFP) and pCL packaging plasmid by the calcium phosphate method. The virus supernatant was collected and used to infect the SKOV3-TR30 cells. The stable cell lines which have decreased expression of miR-17~92 and cell lines transduced by empty plasmids miR-17~92-PTIP-GFP (m-PTIP-GFP) were maintained in the presence of 1 μg/mL doxycycline. Stable transfectants expressing green fluorescent protein were identified by Flow Cytometry and fluorescence microscopy.

### 4.5. Cell Proliferation Assays

Cells were plated at a density of 5 × 10^3^ cells/well in a 96-well plate and incubated with indicated concentrations of paclitaxel (0 nM, 20 nM, 100 nM, 200 nM, quadrupled transfected wells were analyzed for each concentration) for 48 h. The cells were then incubated with MTT reagent and the absorbance at 490 nm was determined.

### 4.6. Cell Cycle Analysis

Cells in the logarithmic phase of growth were selected and paclitaxel were added to the cells with concentration of 0 nM, 20 nM, 100 nM, 200 nM separately. After 48 h, cells were collected and washed in cold phosphate-buffered saline (PBS). After centrifugation at 1000*g* for 5 min, cells were fixed in cold ethanol (70%) over night, washed with cold PBS and stained with 15 μL propidium iodide (50 μg/mL) in the presence of 15 μL of RNase A (10 mg/mL). The cells were incubated for 30 min in the dark and analyzed using Flow Cytometery.

### 4.7. Western Bloting

Cells were collected and washed twice in cold phosphate-buffered saline (PBS). Then cells were solubilized with lysis buffer, on ice for 30 min, and then centrifuged at 12,000 rpm for 15 min at 4 °C. The supernatants were collected, and the protein concentrations were determined using a protein assay CBB kit with a BSA standard.

Samples were loaded for gel electrophoresis at 20 μg/sample, and after electrophoresis, they were blotted onto PVDF membranes. Membranes were blocked for 2 h with blocking buffer at room temperature and then were incubated overnight at 4 °C with rabbit primary antibodies to PTEN and BIM (both from Cell Signaling 1:1000), GAPDH (from Abcam) and then for 1 h at room temperature with HRP-conjugated anti-rabbit (1:2000) or antimouse IgG (1:2000) (both from Abcam). Membranes were then washed three times in TBS-Tween, and specific bands were visualized using the ECL system.

### 4.8. Luciferase Reporter Assays

HEK 293 cells were plated at 2 × 10^5^ cells per well in a 24-well plate 24 h before transfection. The pGL-3-P promoter plasmids containing the wild type or mutated 3′-UTRs of PTEN and 3′-UTRBIM (B1,B2,B3)were co-transfected into HEK 293 cells with TMP-miR-17~92 according to the manufacturer’s instructions using LIPOFECTAMINE 2000, pRL-SV40 (Promega, Madison, WI, USA) was also transfected as a normalization control. Luciferase Assays were performed 24 h after transfection using the Dual Luciferase Assay System. Firefly Luciferase activity was normalized to renilla luciferase activity for each reaction. Quintupled transfected wells were analyzed for each group.

### 4.9. Statistical Analysis

All values are in the expression of mean ± SD; Analysis was done using variance analysis, and comparisons among different groups were made using the Least Significant Difference (LSD) test. It is considered statistically significant when *p* value is less than 0.05.

## 5. Conclusions

In this study, we have demonstrated the significant contribution of miR-17~92/BIM to paclitaxel resistance in human ovarian carcinoma SKOV3-TR30 cells. However, we could not clearly clarify if there is a link from miR-17~92 and PTEN to paclitaxel resistance mechanisms. There are studies to show that decreased PTEN expression is partly responsible for ovarian carcinoma drug resistance [13]. In this present study, however, PTEN was not over-expressed in the resistant SKOV3-TR30 cells after transduced with miR-17~92-PTIP-Sponge all plasmid. Further studies to clarify the possible involvement of other miRNAs that might regulate the expression of PTEN in SKOV3-TR30 cells and the detailed molecular mechanism are needed in our future research.

## Figures and Tables

**Table 1:** Expression level of miR-17~92 in Different Groups.

Cells line	Δct	ΔΔct	2-ΔΔct
SKOV3	9.85 ± 0.198	0.845 ± 0.198	0.55 (0.483–0.642) *
SKOV3-TR30	10.695 ± 0.488	0.0 ± 0.488	1.0 (0.711–1.412)
SKOV3-TR30-m-PTIP-GFP	9.715 ± 0.007	0.98 ± 0.007	0.507 (0.642–0.801) *
SKOV3-TR30-m-PTIP-Sponge all	7.085 ± 0.304	3.61 ± 0.304	0.082 (0.075–0.09) *

* represents compared with the expression level of miRNA-17~92 in SKOV3, the different expression level of miRNA-17~92 in SKOV3-TR30, SKOV3-TR30-m-PTIP-GFP and SKOV3-TR30-m-PTIP-Sponge all is of great significance, *p* < 0.05.

**Table 2:** Relative Content of BIM Protein in SKOV3-TR30 and SKOV3 cells ( *x* ± s).

Cell group	BIM/actin
SKOV3-TR30	0.2118 ± 0.0923
SKOV3	0.2735 ± 0.1233

(*t* = 3.983, *p* = 0.028).

**Table 3:** Relative Content of BIM and PTEN Protein in Each Cell Group (Relative Gray Rate, *x* ± s).

Concentrations of Paclitaxel (nM)	BIM/GAPDH		PTEN/GAPDH	
	m-PTIP-GFP	m-PTIP-Sponge all	m-PTIP-GFP	m-PTIP-Sponge all
0	70.59 ± 3.4215	107.70 ± 2.3265 *	122.77 ± 2.3265	137.67 ± 1.2452
20	94.13 ± 5.3265	154.08 ± 2.2641 *	98.15 ± 5.8736	121.96 ± 1.4737
100	123.72 ± 3.1749	136.83 ± 1.5821 *	122.59 ± 3.5521	130.93 ± 1.2481
200	109.54 ± 1.1399	164.46 ± 3.7326 *	116.54 ± 1.3357	125.28 ± 3.2158

* represents under the same concentration of paclitaxel, the group infected by m-PTIP-Sponge all inhibit plasmid *vs.* the group infected by m-PTIP-GFP empty plasmid, *p* < 0.05.

**Table 4:** Relative Content of PTEN Protein in SKOV3-TR30 and SKOV3 cells ( *x* ± s).

Cell group	PTEN/actin
SKOV3-TR30	0.4043 ± 0.1266
SKOV3	0.4262 ± 0.1422

(*t* = 2.81, *p* = 0.067).
